# Integrin-Associated Protein Promotes Neuronal Differentiation of Neural Stem/Progenitor Cells

**DOI:** 10.1371/journal.pone.0116741

**Published:** 2015-02-23

**Authors:** Kazuhiko Fujimura, Tetsuhiro Niidome, Yoriko Shinozuka, Yasuhiko Izumi, Takeshi Kihara, Hachiro Sugimoto, Akinori Akaike, Toshiaki Kume

**Affiliations:** 1 Department of Pharmacology, Graduate School of Pharmaceutical Sciences, Kyoto University, Kyoto, Japan; 2 Department of Neuroscience for Drug Discovery, Graduate School of Pharmaceutical Sciences, Kyoto University, Kyoto, Japan; 3 World-Leading Drug Discovery Research Center, Graduate School of Pharmaceutical Sciences, Kyoto University, Kyoto, Japan; 4 Graduate School of Pharmaceutical Sciences, Nagoya University, Nagoya, Japan; Hokkaido University, JAPAN

## Abstract

Neural stem/progenitor cells (NSPCs) proliferate and differentiate depending on their intrinsic properties and local environment. During the development of the mammalian nervous system, NSPCs generate neurons and glia sequentially. However, little is known about the mechanism that determines the timing of switch from neurogenesis to gliogenesis. In this study, we established a culture system in which the neurogenic potential of NSPCs is decreased in a time-dependent manner, so that short-term-cultured NSPCs differentiate into more neurons compared with long-term-cultured NSPCs. We found that short-term-cultured NSPCs express high levels of integrin-associated protein form 2 (IAP2; so-called CD47) mRNA using differential display analysis. Moreover, IAP2 overexpression in NSPCs induced neuronal differentiation of NSPCs. These findings reveal a novel mechanism by which IAP2 induces neuronal differentiation of NSPCs.

## Introduction

Neural stem/progenitor cells (NSPCs) possess the ability to self-renew and generate both neuronal and glial lineages. Recent studies have revealed that NSPCs exist not only in the developing brain but also in the subventricular zone (SVZ) and subgranular zone (SGZ) of the adult mammalian brain, including the human brain [1,2]. These findings suggest the possibility of developing NSPC-based therapy for central nervous system (CNS) disorders [3,4].

During CNS development, NSPCs generate neurons and glia sequentially. Emerging evidence indicates that the proliferation and differentiation of NSPCs are regulated by the combination of their cell-intrinsic properties and the local environment. In particular, appropriate early neurogenesis requires receptor tyrosine kinase (RTK)-mediated activation of the MEK-ERK-C/EBP pathway [5], whereas later onset of astrocyte formation requires activation of the JAK–STAT pathway by neuron-derived cardiotrophin-1 [6]. Among local environmental cues, it has been recognized that Delta–Notch signaling is involved in cell–cell interaction and plays an important role in determining the fate of NSPCs [7]. In addition, notch signaling effector, CBF1/RBP-J, directly activates the transcription of astrocytic genes [8]. However, studies on the intracellular signaling cascades linking extracellular signals to transcription in NSPCs are still inadequate.

Integrin-associated protein (IAP; so-called CD47) spans multiple membranes with an amino-terminal extracellular sequence consisting of a single IgV-like domain [9]. It has been recognized that IAP plays an important role in cell–cell contact via several types of ligands, such as signal regulatory protein alpha (SIRPα) [10]. Ligation of SIRPα by IAP promotes tyrosine phosphorylation in the cytoplasmic region of SIRPα[11] and its subsequent association with Src homology 2 domain-containing protein-tyrosine phosphatase 2 (Shp2), resulting in Shp2 activation [12].

In this study, we found that IAP2 promotes neuronal differentiation of NSPCs. First, to investigate the key factors involved in NSPC cell-fate determination, we prepared NSPCs by the neurosphere method and demonstrated that long-term-cultured NSPCs exhibited less neurogenic potential than those cultured for short periods. Second, differential display analysis revealed that short-term-cultured neurospheres expressed high levels of IAP2 mRNA. Finally, IAP2 overexpression in NSPCs significantly increased neuronal differentiation of short-term-cultured NSPCs.

## Materials and Methods

### NSPC cultures

The use of experimental animals in this study was conducted in accordance with the recommendations in the Guiding Principles for the Care and Use of The Japanese Pharmacological Society. Our study was approved by the Kyoto University Animal Experimentation Committee. (Approval Number: 2007–35, 2008–25, 2009–18, 2010–13 and 2011–17). We made all efforts to minimize the number of animals and to limit experiments to necessary to produce reliable scientific information. Primary neurospheres were obtained from SVZ of embryonic day 16 fetal Wistar rats (Nihon SLC, Shizuoka, Japan), as described previously [13]. Briefly, primary neurospheres were incubated for 7 or 14 days. Thereafter, both of them were dissociated and incubated in DMEM/F12 (1:1) (Sigma-Aldrich, St Louis, MO) supplemented with B27 (without Vitamin A) (Invitrogen, Carlsbad, CA), 25 ng/mL recombinant human epidermal growth factor (Peprotech EC, London, UK), 25 ng/mL recombinant human basic fibroblast growth factor (Peprotech), and 5 ng/mL heparin sulphate (Seikagaku Corp., Tokyo, Japan) (NSPC proliferation medium) for 6 days to form secondary neurospheres. Thus, neurospheres were incubated for a total of 13 days *in vitro* (DIV) or 20 DIV. Secondary neurospheres were dissociated and cultured on poly l-lysine-coated dishes in DMEM/F12 (1:1) supplemented with N2 (Invitrogen), penicillin–streptomycin (Invitrogen), and 0.5% FCS (NSPC differentiation medium). After 24 hours, NSPCs were allowed to differentiate in NSPC differentiation medium for 10 days.

### Immunocytochemistry

Cells were fixed with phosphate-buffered saline (PBS) containing 4% paraformaldehyde, washed with PBS, and blocked with 5% normal goat serum (Vector Laboratories Inc., Burlingame, CA) in PBS. Cultures were then incubated at 4°C overnight with primary antibodies diluted in PBS containing 1% normal goat serum. The primary antibodies included mouse monoclonal anti-neuronal class III β-tubulin IgG (Tuj1; 1:500; COVANCE, Berkeley, CA), rabbit polyclonal anti-GFAP (1:1000; DakoCytomation, Glostrup, Denmark), and rat monoclonal anti-GFP (1:1000; NACALAI TESQUE, Inc., Kyoto, Japan). Cells were then incubated for 90 min at room temperature with secondary antibodies diluted in PBS containing 1% normal goat serum. The secondary antibodies included Cy^TM^2-conjugated AffiniPure goat anti-mouse IgG (H + L) (1:1000; Jackson ImmunoResearch Laboratories, West Grove, PA), Cy^TM^2-conjugated AffiniPure goat anti-rat IgG (H + L) (1:1000; Jackson ImmunoResearch Laboratories), Cy^TM^3-conjugated AffiniPure goat anti-mouse IgG (H + L) (1:1000; Jackson ImmunoResearch Laboratories), and Cy^TM^3-conjugated AffiniPure goat anti-rabbit IgG (H + L) (1: 1000; Jackson ImmunoResearch Laboratories). Cells were counterstained with 4′, 6′-diamidino-2-phenylindole (DAPI) (Molecular Probes, Eugene, OR). Immunoreactive cells were quantified in at least four independent experiments. For each experiment, immunoreactive cells were counted in eight randomly chosen fields under 200× magnification, and the results were expressed as a percentage of the total number of cells within the same field. Individual nuclei were stained with DAPI was used to determine the total number of cells per field. Labeled cells were visualized and photographed using an Olympus IX81 photomicroscope (Olympus Optical, Tokyo, Japan).

### PCR differential display

Total RNA was isolated using ISOGEN (Nippon Gene Co., Ltd., Tokyo, Japan) according to the manufacturer’s instructions. PCR differential display analysis was performed using the Fluorescence Differential Display Kit Fluorescein Version (TAKARA BIO INC., Shiga, Japan) according to the manufacturer’s instructions.

### RT-PCR and Real-time PCR

Total RNA was isolated using ISOGEN (Nippon Gene Co., Ltd.) according to the manufacturer’s instructions. Total RNA (2 μg) was reverse-transcribed using a mixture of primers including oligo (dT) primer and the PrimeScript^TM^ RT Reagent Kit (TAKARA BIO INC.). RT-PCR was performed as previously described [13]. Forward (F) and reverse (R) PCR primers for IAP were designed against rat transcripts as follows: F, 5′-GATGGCTTTCGCAGAGCAAAC-3′ and R, 5′-TCTGATGAGAAGAAATGACACG-3′. RT-PCR conditions were 2 min at 95°C, followed by 40 cycles of 30 s at 95°C, 30 s at 55°C, and 30 s at 72°C. Real-time reverse transcription PCR was performed using SYBR Premix EX Taq^TM^ Kit (TAKARA BIO INC.). The pairs of primers for rat IAP2 mRNA were designed using ProbeFinder software (http://www.roche-applied-science.com), and the nucleotide sequences of the forward and reverse PCR primers used were 5′-CAGCGAACTATACAAC-3′ and 5′-CGTAAATTACAGCTGC-3′, respectively. The pairs of primers for rat peptidylprolyl isomerase A (Ppia, RA015371) mRNA were purchased from TAKARA BIO INC. PCR amplification and fluorescence detection were performed using the Thermal Cycler Dice Real Time System (TP850, TAKARA BIO INC.). All of the real-time PCR conditions were 10 s at 95°C, followed by 40 cycles of 10 s at 95°C, 10 s at 50°C, and 10 s at 72°C.

### Retroviral infection

Rat IAP2 cDNAs were amplified by PCR from rat NSPC cDNA and their coding regions were verified by DNA sequencing. This cDNA was subcloned into pMCs-IRES-EGFP [a replication-incompetent retroviral vector containing an internal ribosome entry site (IRES) sequence followed by the coding sequence for enhanced GFP] (CELL BIOLABS, INC., San Diego, CA). The pMCs-IRES-EGFP vector was used for the production of recombinant retroviruses. Ecotropic virus-packaging (PLAT-E) cells (CELL BIOLABS, INC.) were transfected with the desired plasmids using FuGENE 6 (Roche) and then cultured for 3 days at 37°C. Retroviral particles were collected from the culture supernatant by centrifugation at 15000 *g* for 5 hours at 4°C and resuspended in NSPC proliferation medium. For retroviral infection, 7 DIV or 14 DIV primary neurospheres were dissociated and mixed with recombinant retrovirus in Ultra-Low Attachment 6 well dish (Corning, INC., Corning, NY). After 24 hours, NSPCs were resuspended in proliferation medium and cultured for 6 days in poly hydroxyethyl methacrylate (Sigma-Aldrich)-coated 10-cm culture dish.

### Western blot analysis

Western blot analysis was conducted as described previously [13] using mouse monoclonal anti-IAP (CD47) antibody (1:500; AbD Serotec Ltd., Oxford, UK) or mouse monoclonal anti-glyceraldehyde-3-phosphate dehydrogenase (GAPDH) antibody (Ambion, Austin, TX), followed by horseradish peroxidase-conjugated sheep anti-mouse IgG antibody (1:2000; Amersham Biosciences, Buckinghamshire, UK). Bands were visualized by enhanced chemiluminescence (ECL detection kit; Amersham Biosciences).

### Statistical analyses

Each value represents the mean ± SEM. Statistical comparisons were made using Student’s *t*-test or one-way analysis of variance, followed by Bonferroni’s multiple comparison test using the SPSS version 12.0 program (SPSS Inc., Chicago, IL). Results were considered significant at *p* < 0.05.

## Results

### In vitro time-dependent decline of neurogenic potential of NSPCs

**Fig 1:**
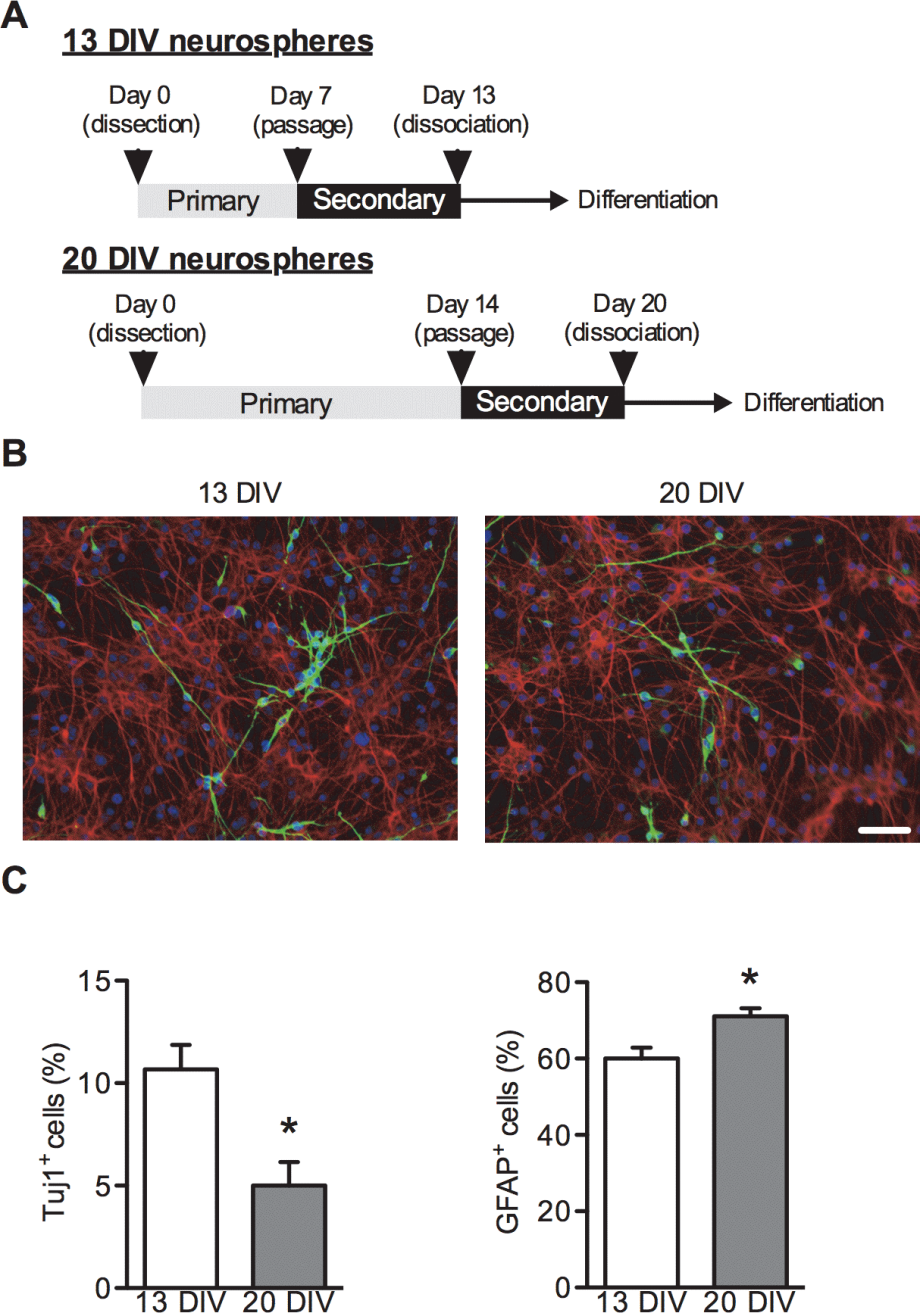
*In vitro* time-dependent decline in neurogenic competency of NSPCs. **(A)** Strategy for the functional screening of candidate genes involved in the temporal specification of NSPCs using the neurosphere method. (B) Representative immunofluorescent images of NSPCs differentiated from 13 DIV neurospheres (left) and 20 DIV neurospheres (right). Secondary neurospheres were dissociated in NSPC proliferation medium and plated on poly l-lysine-coated cover glass. After 24 hours, NSPCs were exposed to NSPC differentiation medium, fixed at 10 days after differentiation, stained for a neuron marker (Tuj1; green) and an astrocyte marker (GFAP; red), and counterstained with DAPI (blue). Scale bar: 50 μm. Quantification of Tuj1-positive cells (C) or GFAP-positive cells (D) 10 days after differentiation. Data are shown as the mean ± SEM of four independent experiments. *p < 0.05.

NSPCs were obtained from SVZ of rats on embryonic day 16 and cultured in NSPC proliferation medium as floating neurospheres [13,14]. Because NSPCs switch their differentiation potency from neurogenic to gliogenic in a time-dependent manner *in vivo* and *in vitro* [6], we prepared 13 DIV and 20 DIV neurospheres as described in the Methods and as shown in Fig. 1A. Immunocytochemistry showed that the percentage of Tuj1-positive cells differentiated from 20 DIV neurospheres significantly decreased compared with that from 13 DIV neurospheres (Fig. 1B, C). In contrast, the percentage of GFAP-positive cells differentiated from 20 DIV neurospheres was increased compared with that from 13 DIV neurospheres (Fig. 1B, D). These data indicate that, in our culture system, long-term-cultured NSPCs temporally lose neurogenic potential and obtain gliogenic potential.

### Expression of IAP2 mRNA in NSPCs

**Fig 2:**
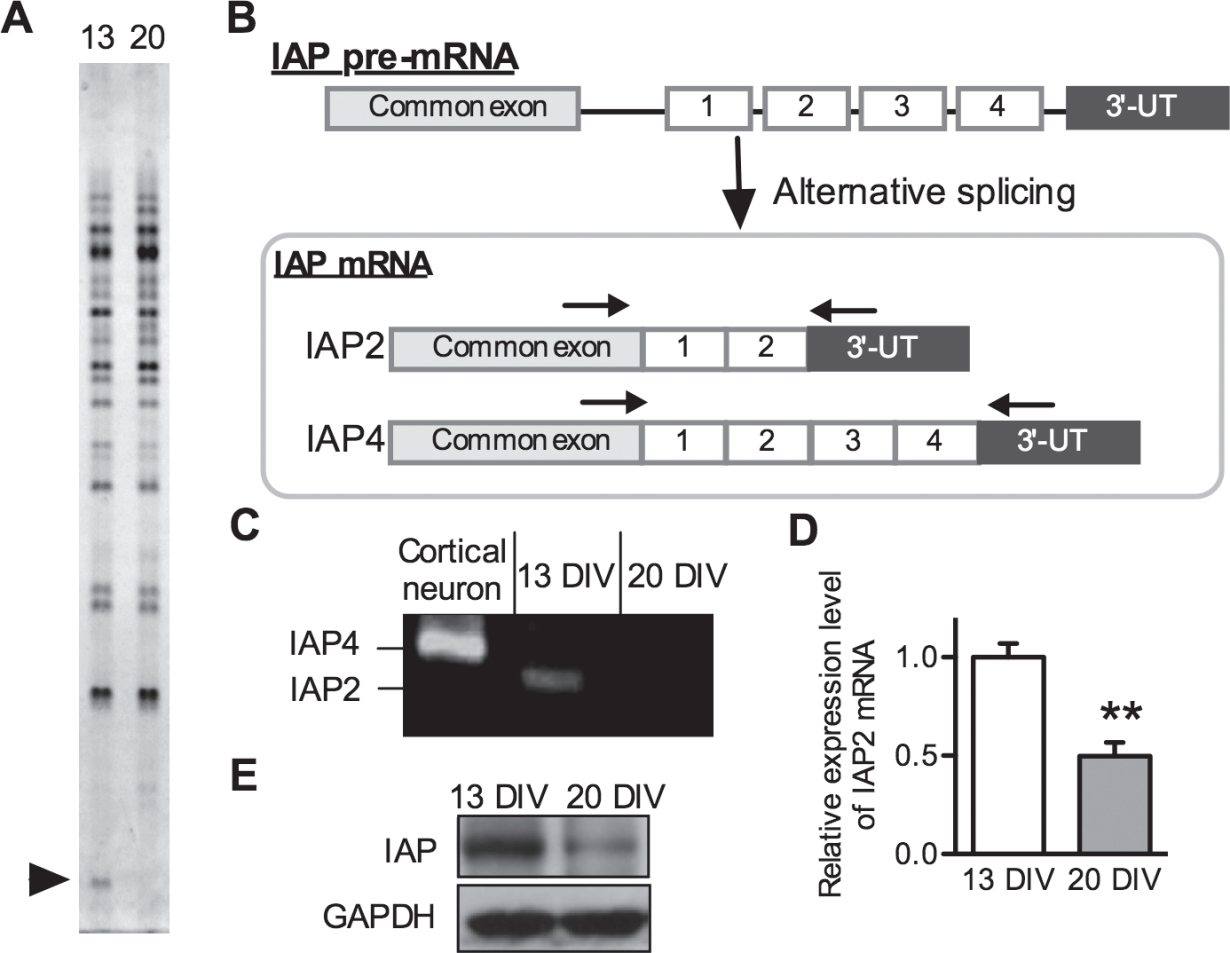
Expression of IAP2 mRNA in NSPCs. (A) Differential display analysis of 13 DIV neurosphere (left lane) and 20 DIV neurosphere (right lane) RNA using No. 22 upstream primer and 5′-T_n_AC-3′ downstream primer sets. Arrowhead indicates the differentially expressed IAP2 cDNA fragment between 13 DIV neurospheres and 20 DIV neurospheres. (B) Schematic representation of the alternative splicing possibilities for generation of IAP forms. Alternative splice forms of IAP were identified by RT-PCR analysis using primer pairs designed in common exon sequences (arrows). Right arrow indicates the position of the forward primer and the left arrow indicates the position of the reverse primer for RT-PCR as shown in (C). (C) Identification of splicing forms of IAP expressed in NSPCs using RT-PCR analysis. Total RNA was isolated from 13 DIV neurospheres and 20 DIV neurospheres. Alternative splice forms were distinguished by the size of PCR products using primer sets shown in (B). (D) Quantification of IAP2 mRNA expression levels in 13 DIV neurospheres and 20 DIV neurospheres. Total RNA was isolated from 13 and 20 DIV neurospheres. The expression levels of IAP2 and Ppia (internal standard) mRNA were determined by real-time quantitative PCR analysis. (E) Western blot analysis of IAP protein expression at 13 DIV (left lane) and 20 DIV (right lane). Each value represents the mean ± SEM from three independent experiments and is expressed in reference to 13 DIV neurospheres. **p < 0.01 compared with 13 DIV neurospheres.

To identify differences in gene expression between 13 DIV and 20 DIV neurospheres, we performed fluorescence PCR differential display analysis [15]. Total RNA samples extracted from 13 DIV and 20 DIV neurospheres were subjected to PCR differential display analysis using a fluorescence differential display kit (TAKARA BIO, Inc.). Most of the cDNA bands were identical among individuals; however, one cDNA band was differentially expressed between 13 DIV and 20 DIV neurospheres, when 5′-T_n_AC-3′ downstream primer and No.22 (5′-AGCCAGCGAA-3′) upstream primer were used as the primer pairs (Fig. 2A). This sequence was 100% homologous to the 3′-end region of rat IAP cDNA (GenBank ID: NM019195.2). IAP is a membrane protein and its cytoplasmic tail possesses four alternative splicing forms [9,16] (Fig. 2B). It has been reported that form 4 is mainly expressed in the adult mouse brain [16]; however, the isoform expressed in NSPCs and the role of IAP in NSPCs is unknown. To determine the alternatively spliced forms in NSPCs, we performed RT-PCR on NSPCs using oligonucleotides that were present in all 4 forms of IAP and bracketed the region of the mRNA encoding the alternatively spliced cytoplasmic tails (Fig. 2B). Using this PCR strategy, the 4 forms could be distinguished based on the size of the PCR products (Fig. 2B, C). This RT-PCR analysis revealed that NSPCs predominantly expressed form 2 IAP (IAP2) mRNA; however, mature neurons expressed form 4 (Fig. 2C). These data indicate that NSPC populations with high expression of IAP2 tend to differentiate into neurons. In addition, real-time RT-PCR showed that 13 DIV neurospheres had approximately 2-fold higher levels of IAP2 mRNA expression than 20 DIV neurospheres (Fig. 2D), which is consistent with the results of the differential display analysis. Western blot analysis demonstrated that the protein expression level of IAP was also higher in 13 DIV neurosphere than 20 DIV neurosphere (Fig. 2E). These data suggest that IAP2 is involved in the mechanisms by which NSPCs change their neurogenic and gliogenic potential in a time-dependent manner.

### Effect of IAP2 overexpression on NSPC differentiation

**Fig 3:**
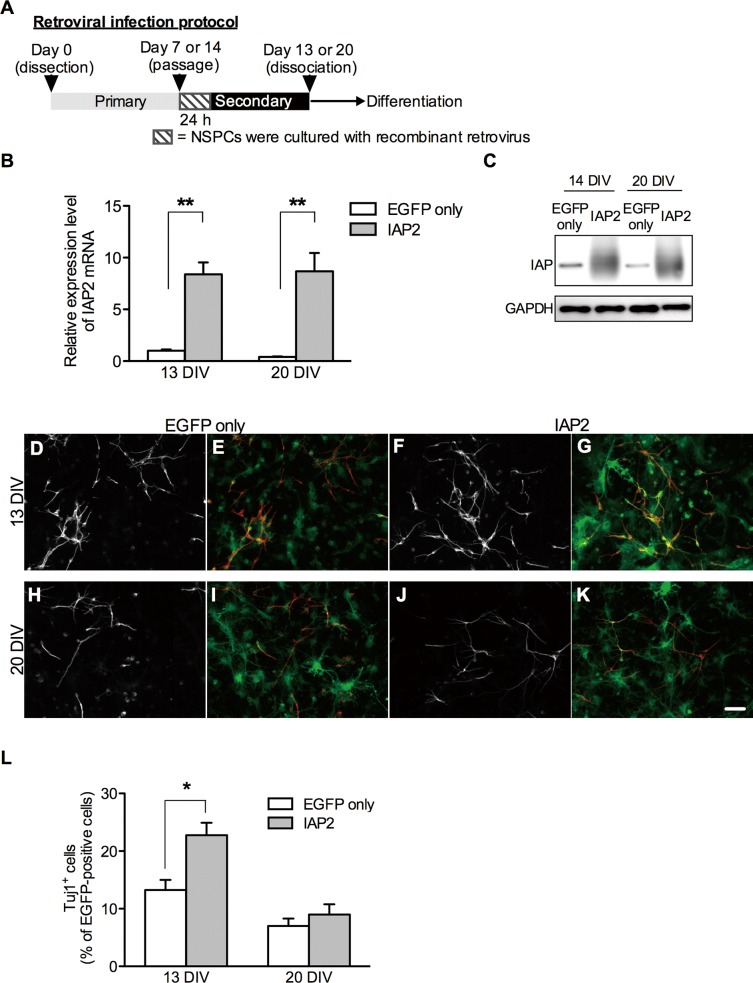
Effects of IAP2 overexpression on NSPC differentiation. (A) Schematic diagram illustrating experimental design followed throughout this study. Primary neurospheres were dissociated and infected with a retrovirus encoding EGFP only or EGFP and IAP2 (IAP2). After 24 hours, cells were washed and resuspended in proliferation medium to form secondary neurospheres. Confirmation of IAP2 overexpression by real-time PCR (B) and western blot (C) in 13 DIV and 20 DIV neurospheres. After retrovirus-infected secondary neurospheres were cultured for 6 days, 13 DIV neurospheres (D-G) and 20 DIV neurospheres (H-KE–H) were dissociated and harvested as Fig. 1. Cells were fixed on day 10 after differentiation and immunostained for a neuronal marker (Tuj1; D, F, H, J). Immunofluorescent images of cells stained for EGFP (green) were merged to images of cells stained for Tuj1 (red; shown in E, G, I, K). Scale bar for all: (in K) 50μm. (L) Quantification of Tuj1-positive cells (expressed as a percentage of EGFP-positive cells). Data represent the mean ± SEM from the four independent experiments. *p < 0.05.

To study the role of IAP2 in NSPC differentiation, we infected secondary neurospheres with a retrovirus encoding the rat IAP2 open reading frame and harboring enhanced green fluorescence protein (EGFP) or EGFP only (control) (Fig. 3A). We confirmed IAP2 overexpression by real-time PCR (Fig. 3B) and western blot analysis (Fig. 3C). The percentage of Tuj1-positive cells was significantly increased when IAP2 was overexpressed in 13 DIV neurospheres (Fig. 3D-G, L). On the other hand, there was no significant change in the percentage of Tuj1-positive cells when IAP2 was overexpressed in 20 DIV neurospheres (Fig. 3H-K, L). These results suggest IAP2 is specifically expressed in neurogenic NSPCs and can promote neuronal differentiation of short-term-cultured neurogenic NSPCs because IAP2 stimulates neuronal differentiation of young neurogenic NSPCs derived from 13 DIV neurospheres, but long-term-cultured gliogenic NSPCs derived from 20 DIV neurospheres are not reactive to IAP2 stimulation.

## Discussion

Cell-fate determination of NSPCs is regulated by cell-intrinsic mechanisms and local environmental cues. However, the intracellular signaling cascades linking extracellular signals to transcription in NSPCs remain unknown. In this study, we report for the first time that a membrane protein IAP2 induces neuronal differentiation of NSPCs. This conclusion is based on the following results: (i) fluorescent differential display revealed that neurogenic NSPCs expressed higher levels of IAP2 mRNA than gliogenic NSPCs and (ii) immunocytochemical analysis revealed that IAP2 overexpression increased the percentage of Tuj1-positve cells.

In this study, we prepared two distinct populations of NSPCs, which differed in their neurogenic potential. It has been reported that NSPCs change their differentiation potential from neurogenic to gliogenic in a time-dependent manner *in vivo* and *in vitro* [6,17,18]. We demonstrated that 13 DIV neurospheres differentiate into more neurons than 20 DIV neurospheres suggesting that NSPCs gradually decreased their neurogenic potential in our culture system.

IAP gene expresses four alternative splicing variants and the alternative splicing forms of IAP mRNA are tissue specific [16]. Although previous reports showed that IAP4 is mainly expressed in the adult mouse brain [16,19], it was unknown whether IAP is expressed in NSPCs. Furthermore, as none of the IAP cytoplasmic extensions has any known motif for enzymatic activity or protein interaction, the difference in downstream signaling among the 4 splicing forms is unknown. In this study, RT-PCR analysis showed that NSPCs predominantly express IAP2 mRNA and other forms of IAP were not detected, indicating that the expression of IAP2 mRNA could be a marker for neurogenic NSPCs.

Although we demonstrated that overexpression of IAP2 significantly increased the percentage of Tuj1-positive cells differentiated from 13 DIV neurospheres, the downstream signaling for IAP that induces neuronal differentiation of NSPCs is unknown. It has been reported that the interacting protein partners of IAP, such as integrins and SIRPα are important for IAP2-mediated downstream signaling [9,20]. IAP-integrin interaction occurs only within a single membrane. On the other hand, IAP-SIRPα interaction can mediate cell–cell contact. Interestingly, in this study, when IAP2 was overexpressed in 13 DIV neurospheres, the percentage of Tuj1-positive cells differentiated from not only EGFP-positive NSPCs, but also EGFP-negative NSPCs that did not overexpress IAP2, was increased (data not shown), suggesting that IAP2-overexpressing cells induce neuronal differentiation of neighboring cells. These data indicates the possible involvement of the IAP-SIRPα signaling pathway in IAP2-induced neuronal differentiation of NSPCs.

Importantly, in the present data, IAP2 did not affect the differentiation of 20 DIV neurospheres with low expression levels of IAP2, despite the fact that 13 DIV neurospheres exhibited neuronal differentiation induced by IAP2 overexpression. Because NSPCs temporally change their neurogenic potential by environmental cues and cell-intrinsic mechanisms [2], it is possible that cell-intrinsic mechanisms, such as epigenetic changes, inhibit differentiation of 20 DIV neurospheres into neurons. Since a previous report showed that neuronal environmental Wnt-β-catenin signaling could not induce neuronal differentiation of NSPCs in the later stages of brain development, in which induction of neurogenin-1 transcription by β-catenin is epigenetically inhibited [21], it may be possible that IAP2-mediated neuronal differentiation is also attenuated in 20 DIV neurospheres by cell-intrinsic mechanisms.

In conclusion, this is the first report to show that IAP2 promotes neuronal differentiation of NSPCs. Our results provide fundamental insights that may be useful to clarify the molecular mechanisms that regulate cell-fate determination of NSPCs.
